# Agile research to complement agile development: a proposal for an mHealth research lifecycle

**DOI:** 10.1038/s41746-018-0053-1

**Published:** 2018-09-13

**Authors:** Kumanan Wilson, Cameron Bell, Lindsay Wilson, Holly Witteman

**Affiliations:** 1 Faculty of Medicine, University of Ottawa, Ottawa, Canada; 2 Clinical Epidemiology Program, Ottawa Hospital Research Institute, Ottawa, Canada; 3 Department of Family and Emergency Medicine, Université Laval, Quebec, Canada

**Keywords:** Medical research, Public health

## Abstract

Mobile health (mHealth) technology is increasingly being used, but academic evaluations supporting its use are not keeping pace. This is partly due to the disconnect between the traditional pharmaceutical approach to product evaluation, with its incremental approach, and the flexible way in which mHealth products are developed. An important step to addressing these problems lies in establishing agile research methods that complement the agile development methodologies used to create modern digital health applications. We describe an mHealth research model that mirrors traditional clinical research methods in its attention to safety and efficacy, while also accommodating the rapid and iterative development and evaluation required to produce effective, evidence-based, and sustainable digital products. This approach consists of a project identification stage followed by four phases of clinical evaluation: Phase 1: User Experience Design, Development, & Alpha Testing; Phase 2: Beta testing; Phase 3: Clinical Trial Evaluation; and Phase 4: Post-Market Surveillance. These phases include sample gating questions and are adapted to accommodate the unique nature of digital product development.

## Introduction

The ongoing expansion of mobile health (mHealth) technology is both an enabler and a consequence of a nascent era of healthcare, one in which patient-centered care, precision medicine, and equitable access form the health system’s prime directives. Given that one-third of individuals in the United States who use technology to manage their health report using a health app,^1,2^ and that global mHealth and digital health revenues are projected to exceed $500B by 2025,^3^ the scale-up of mHealth into an interoperable, standards-based, and commercially viable ecosystem appears overdue. However, the evidence base for mHealth technologies is relatively sparse.^4,5^ Pilot studies of hundreds of applications have yielded little evidence to guide the implementation and scale-up of mHealth technologies, and the industry as a whole has not yet established the standards, technological infrastructure, or comprehensive regulatory frameworks that will be required to realize the ultimate potential of mHealth.^4,5^

These issues are compounded by the rapid pace of change in mobile technology and the multi-faceted nature of new mobile health apps and interventions, which may be significantly more complex than the SMS-based interventions on which mHealth research has been primarily based in the past.^6^ We believe that an important step to addressing these problems lies in establishing agile research methods that complement the agile development methodologies used to create modern digital health applications. Agile methods emphasize ongoing and iterative development based on continuous feedback. We describe an mHealth research model that mirrors the traditional approach to pharmaceutical or clinical intervention research in its attention to safety and efficacy, while also accommodating the rapid and iterative development and evaluation required to produce effective, evidence-based, and sustainable digital products. We believe this approach will offer guidance to individuals considering different strategies for regulating digital products and to individual and system-level decision-makers regarding widespread product adoption. This approach could improve the quality and safety of digital health solutions by facilitating ongoing evaluation, an element of particular relevance for academic developers seeking to simultaneously create and evaluate digital products. Such an approach may also promote industry-academic partnerships to combine development with more rigorous product evaluation.

## Existing strategies for evaluating digital health products

Given the widespread use of digital health products, several approaches have been proposed for how best to combine research with development in the digital space. One notable example is the IDEAS framework, which combines strategies from behavioral theory, design thinking, user-centered design, evaluation, and dissemination to develop a 10-step, 4-stage process that can be utilized in the development of digital health interventions.^7^ By combining methods from a variety of disciplines, this approach seeks to guide the development of more effective digital tools for changing health behaviors.

In addition, in light of the particular challenges associated with implementing and reporting on online health interventions, including issues with blinding and capturing sufficient study details for replicability, in 2010 the CONSORT-EHEALTH checklist was developed. This checklist acts as an extension of the CONSORT statement, which was developed to improve the reporting of randomized control trials. The E-HEALTH checklist adapts this approach to eHealth and mHealth interventions, recommending best practices for reporting and knowledge translation that accommodate the unique nature of digital health research.^8^

Recently, an assessment conducted by a working group organized by the Mobile World Capital Barcelona Foundation sought to explore existing mHealth assessment initiatives and their potential effectiveness.^9^ The authors note that developers should be engaged in the product evaluation process and provided with guidance regarding how to make products safe and effective in order to ensure the evaluation process is mutually beneficial for both developers and users. The authors also recommend that all relevant stakeholders be engaged in a continuous, iterative evaluation process built on a common foundation of guidelines and recommendations.^9^

## Regulatory strategies

Coinciding with the development of these academic approaches to improved mHealth regulation and evaluation, governmental agencies have also been developing strategies to more effectively regulate digital health products. In the United States, the Food and Drug Administration (FDA) has conducted extensive work to balance the importance of rapid innovation with the need to regulate mHealth products that may be classified as medical devices. In 2015, non-binding recommendations were issued by the FDA to provide guidance to developers and manufacturers of mobile applications that would be considered medical devices and could present a risk to safety if they did not function as intended.^10,11^ Using a risk-based approach, FDA oversight was recommended for all mHealth products considered to be high-risk. More recently, it has been suggested that rather than regulating individual devices, eligible developers could qualify for pre-certification by demonstrating a track-record of high-quality products, allowing their digital solutions to undergo a faster and more streamlined evaluation process.^12^ These pre-certified developers could also make use of the National Evaluation System for health Technology (NEST), a system that promotes innovation by generating real-world evidence across the total product lifecycle.^12^

In Canada, efforts are also underway to develop new approaches for regulating digital health technologies.^13^ In April 2018, Health Canada announced the establishment of a new Digital Health Review Division, intended to promote pre-market review of mHealth technologies while accommodating rapid development and innovation. This addition is part of ongoing efforts on the part of Health Canada to improve the regulatory system currently in place for mHealth products.^13^

## Barriers to use

The primary challenge to the application of any evaluation framework is the potential perception from developers that resources should not be invested in research because such efforts will slow down innovation (a perception that has also been acknowledged by the FDA^14^). However, as regulatory processes mature and move toward legislation, some form of data on digital products’ effectiveness and safety, particularly those products that are viewed as medical devices, will be increasingly required.^10,15^ Meshing research and development as seamlessly as possible will help create the capacity to meet these regulatory requirements. At the same time, it will help developers to improve and continuously iterate their solutions based on research evidence.

## mHealth’s incongruence with traditional research models

**Fig. 1:**
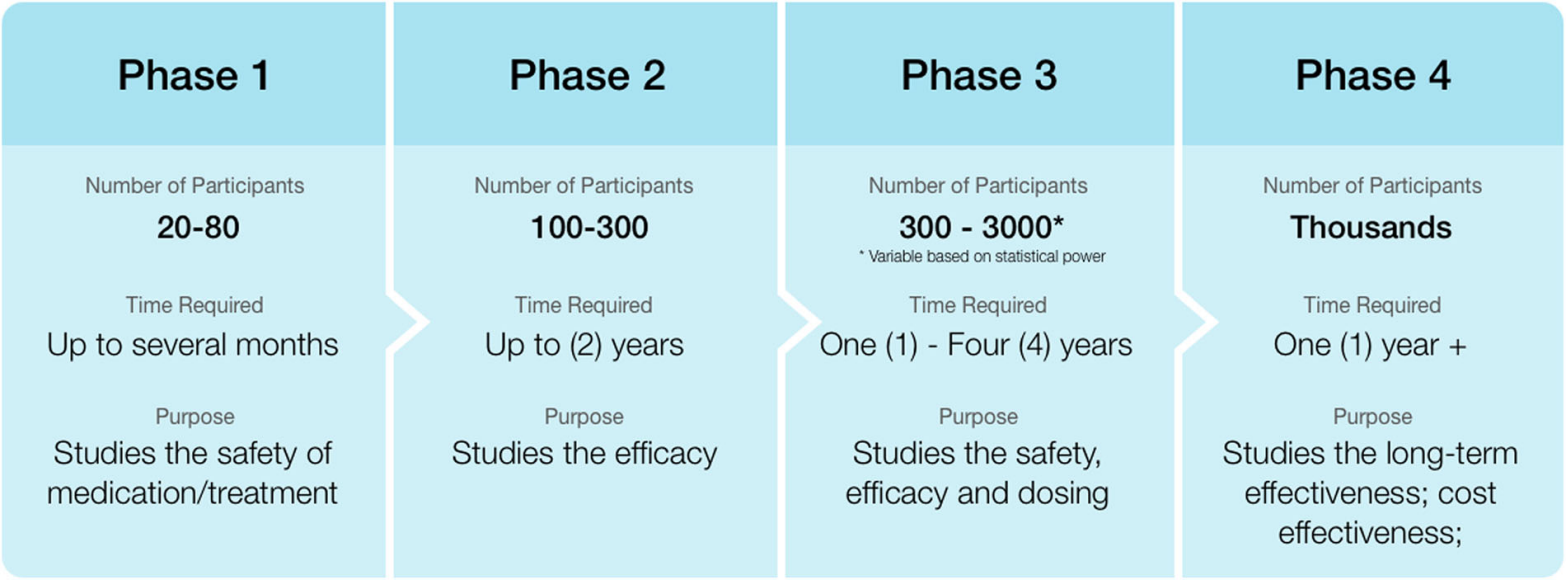
The traditional four-phase pharmaceutical evaluation framework. (Adapted from K. Sagitova, 2009. Watching clinical trials liability. *Risk Futures*.)

The approaches described above recognize the uniqueness of the total product life cycle and the ongoing evolution of medical devices.^16^ Pharmaceutical products have a well-described path to market consisting of four phases of clinical evaluation (Fig. 1).^17^ This approach sequentially tests a product to determine in sequence its safety, efficacy, and effectiveness. This path to market is linear and typically not modifiable; if a product fails in early phase studies, there is usually no opportunity to make changes that would permit moving forward to later phase studies. By contrast, the development of digital health products is non-linear. Dynamic and iterative approaches are required for a number of reasons. First, the speed at which consumer digital technology evolves necessitates a considerably faster development process than standard clinical interventions. Second, linear evaluation processes to establish drug safety can take upwards of 10 years, while digital products can be iteratively enhanced and modified almost immediately following identification of system bugs and upon receipt of user feedback. Both of these elements can allow for a development and evaluation process that is much faster than the clinical trial approach used in pharmaceutical research.

**Fig. 2:**
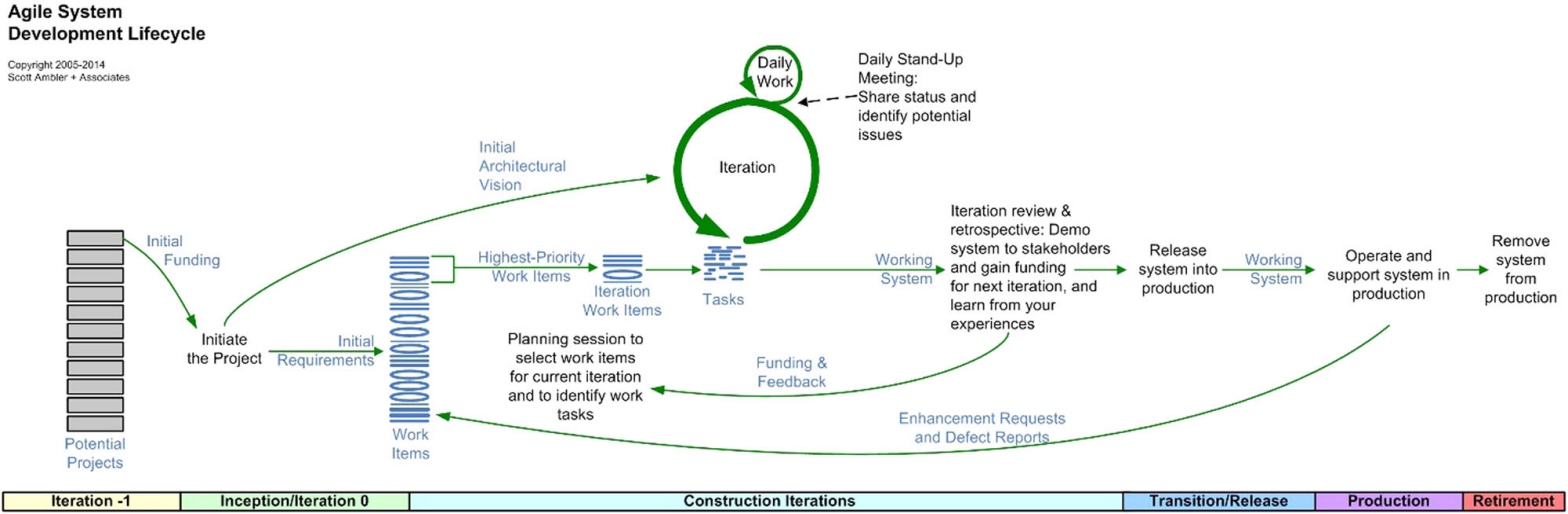
The Agile System Development Lifecycle, courtesy of Ambler 2008 (ref^32^)

One common approach to digital product development is Agile Development (Fig. 2), which seeks to provide software at regular short intervals, offers flexibility to respond to changing requirements, and incorporates the ability to adapt to feedback through continuous iteration. Ideally, agile development emphasizes face-to-face collaboration^18^ and interactions between developers and customers as the best mechanism to create effective solutions for the end-user.^19^

However, in the development of mHealth products that present more than a minimal risk to users, as in the development of pharmaceuticals, safety and efficacy evaluations are required by regulatory agencies, necessitating a development process that supports such evaluations.^10,15^ For pharmaceuticals, the traditional multi-phase clinical trials approach has proven to be a successful way of developing safe and effective medications. This methodology serves as a gating process, only allowing through products with sufficient evidence to support their use. Likewise, mHealth apps could benefit from a similar gating process. However, unlike pharmaceuticals, the gating process for mHealth apps can facilitate additional iteration of the product at each impasse, until the product either succeeds and progresses to the next phase or is deemed unviable and is retired.

Mobile products are also different from pharmaceuticals in that apps often have many features or components that can exist at different phases of development simultaneously, while individual components of a drug cannot usually be significantly altered during the pharmaceutical clinical trial process. This is especially true for complex mHealth products which may incorporate a broad set of features and interventions into a single platform.

Finally, neither agile nor traditional pharmaceutical development fully incorporate methods that ensure a developed product is well-designed for the people who will use it. Methods that do so are found in related approaches and traditions often grouped under labels such as user-centred design, human-centred design, user experience design, co-design, or design thinking.

## An agile and user-centred research and development model

The aforementioned digital product regulation strategies proposed by academics and regulatory officials will be important components of the eventual success of the digital health space. As strategies for mHealth app regulation move toward legislation, we recommend a development approach that combines the agile development process with current methods used to evaluate pharmaceuticals. We believe that this approach can help developers build better products while also preparing them to meet regulatory requirements.

**Fig. 3:**
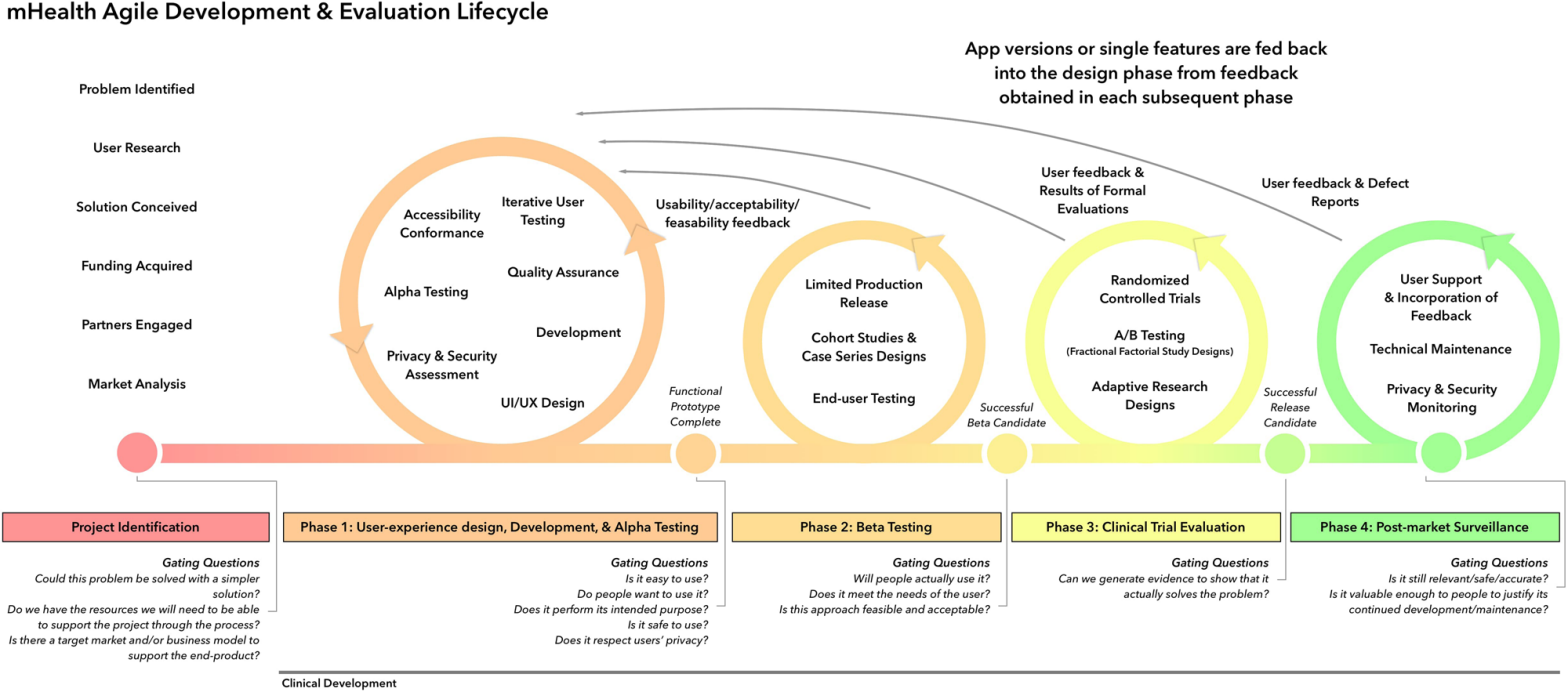
mHealth Agile Development & Evaluation Lifecycle

We contend that elements of both agile development and traditional pharmaceutical research may be integrated to create a new “mHealth Agile and User-Centred Research and Development Lifecycle”. We propose a framework that applies a four-phase approach to mHealth product development and evaluation in order to produce high-quality apps that solve real problems while also being safe, effective, and commercializable. Figure 3 presents our proposed lifecycle. This proposed framework incorporates a similar set of steps to those currently in use by the FDA in the approval of medical devices,^20^ but modifies this approach in order to allow for continuous, iterative development. Using a risk-based approach similar to that used by the FDA, many products will require only Phase 1 or Phase 2 evaluation, and for many products, individual elements of the product can be evaluated while an earlier version is already on the market. For each phase, we have identified a set of sample gating questions that can facilitate the decision regarding whether a product should advance to the next stage in the development cycle. These questions are not exhaustive, as certain questions will not be relevant in some cases while additional factors may need to be considered in other cases. These questions are instead intended to encourage thought and discussion about a product’s practical viability at all stages of development.

Importantly, our approach promotes the development of high-quality, thoroughly-tested applications and is aligned with the workflows that have been built into app publishing platforms (e.g., Apple’s App Store; Google Play) to promote a high level of quality and performance.^21^ Our framework’s alignment with these workflows will ideally both encourage its use by developers and facilitate the product’s evaluation by the publisher.

### Project identification phase

Sample Gating Questions: Does this problem actually require a digital platform or could it be solved with a simpler solution? Do we have the expertise and resources we will need to be able to support the project through the process? Is there a target market and/or business model to support the end-product?

In this stage, a challenge is identified, a product is envisioned, and a project is initiated. Team members are identified, funding is secured, and initial requirements are established. The idea for the product is evaluated against other approaches to solving the identified problem. The vision for the product is also evaluated based on its ability to attract funding and talent with which to undertake the project. It is important that a long-term view is taken when evaluating whether the project will have sufficient resources to make it through the mHealth development and evaluation lifecycle. There would ideally also be an identified target market for the product so that financial sustainability for the project is conceivable.

### Clinical development

#### Phase 1: User experience design, development, & alpha testing

Sample Gating Questions: Is it easy to use? Do people want to use it? Does it perform its intended purpose? Is it safe to use? Does it respect users’ privacy? Is it accessible to people at various levels of ability? Do people enjoy using it?

Once the project has reached the development phase, the first step is to develop and evaluate the user experience. Proposed designs, conceived as storyboards and wireframes, may be evaluated using iterative user testing/user-interface testing. This process ensures that the product design incorporates an understanding of the user’s needs, values, context, abilities, strengths, and limitations.^22^ The design can then undergo iteration until the above criteria are met.

While the design is being refined, development can begin, resulting in an initial functional prototype. This prototype then enters alpha testing, in which test engineers and project team members evaluate the functionality of the product to determine whether it performs its intended purpose. This process helps to eliminate bugs and other critical issues. Early feedback from alpha testers may also inform the design of the product or new features, resulting in further design phase iteration cycles.

It is also during this phase that key assessments are performed to determine whether the product is technically secure and aligned with privacy and accessibility standards. For the former, it is typical to perform threat risk assessments, privacy impact assessments, and technical vulnerability assessments. For the latter, the accessibility of the functional prototype can then be assessed according to Web Content Accessibility Guidelines (WCAG) 2.0 standards.^23^ This determines the extent to which the product can be used without presenting barriers to people with disabilities.

#### Phase 2: Beta testing

Sample Gating Questions: Will people actually use it? Does it meet the needs of the user? Is this approach feasible and acceptable? Can it be implemented in its intended context? Is there sufficient face validity for this product to solve the challenge at hand?

Once a functional prototype is developed, mHealth products can be tested in cohorts of external users, such as patients or members of the public. This beta testing seeks to further determine (in conjunction with data from alpha testing) whether the developed prototype meets the needs of users in ways that will lead to sustained adoption. Here, the acceptability and accessibility of the product can be ascertained and evaluated against patient demographics (age, sex, gender, education, literacy, sociodemographic status, health status, technology readiness)^24,25^ in order to identify any equity issues or barriers to access. This will allow for an exploration of patients’ willingness and ability to use the product, as these will impact upon adherence, a critical component of a product’s effectiveness. This creates an opportunity for evaluation using feedback from users or more formal evaluations based on surveys, interviews, focus groups, and analytics data.

#### Phase 3: Clinical trial evaluation

Sample Gating Questions: Can we generate evidence to show it actually solves the problem?

A successfully-tested beta version of the product may be considered for clinical trial study designs in order to generate evidence that the product solves the clinical problem for which it was originally designed. While “low-risk” general wellness or data access applications do not necessarily require clinical evaluation according to FDA guidlines,^10^ such evaluations may help better inform members of the public seeking benefit from these products and can give these products credibility.^15^ Apps that may be “used in the treatment, mitigation, diagnosis or prevention of a disease or abnormal physical condition” should be carefully tested prior to large-scale implementation. A product that meets this characterization is often classified as a medical device, and, in many countries, is thus subject to regulatory requirements that govern its path to market.^26^ We expect that randomized controlled trials will become a standard requirement in the evaluation of the safety and effectiveness of mHealth products that fall under the definition of a medical device.

“Low-risk” apps (those that do not pose any risk to user safety if they do not function properly^10^) that do not meet the criteria for medical devices may still benefit from clinical trials, but this process does not necessarily need to occur before the product’s public release, as different elements of a product can exist at different stages of development simultaneously. Clinical trials and peer-reviewed evidence demonstrating a product’s efficacy and usefulness are important in establishing the credibility of a product, especially given the flood of mHealth apps on the market, the vast majority of which have not been clinically evaluated.^27^

One form of product evaluation that is commonly used in agile development is called A/B testing. A/B testing has become a mainstay for internet technology companies looking to improve or optimize their products. In A/B testing, a subset of an app’s users is randomized to receive a version of the app with minor variations in order to test which version performs better according to a predetermined metric (e.g., which version leads to optimal clicking of a particular button, use of a particular feature, or overall usage statistics). A/B testing can be used to evaluate aspects of the user experience or clinical functionality, and the results of the testing can then be iterated back through the design/development cycle. As the technological mechanism to support the randomization groups of a clinical research trial, individual A/B tests can also be much smaller in scope and significance, and can occur at any point in the development lifecycle. Many companies employ “continuous experimentation”, through which they may perform many small concurrent A/B tests per week.^28^ Developers of mHealth apps should consider how they plan on employing A/B testing early in the design phase so that the necessary technical infrastructure to support A/B testing can be included from the beginning.

Novel study designs may also be better suited to evaluating mHealth products than standard randomized trials. For example, adaptive research designs may be particularly useful in evaluating multi-faceted interventions such as mHealth products where the functionality can be initially disaggregated and then combined based on the proven effectiveness of each individual component.^29,30^ This methodology also offers the potential to reduce both the sample size and length of a study, key considerations in evaluating digital solutions, by exploiting interim analyses to identify early treatment effects.^31^ We expect that adapting current clinical trial methodology to better fit with the characteristics of an agile development approach will be an area of major focus.

As in the other phases, user feedback and the results of formal evaluations can be fed back into the iteration process to improve the product.

#### Phase 4: Post-market surveillance

Sample Gating Questions: Is it still relevant/safe/accurate? Is it valuable enough to people to justify its continued development/maintenance? Can it be further improved?

Post-market surveillance is standard practice for monitoring the safety of a pharmaceutical drug or medical device after it has been released to the market. This holds true for mHealth products as well. Once a product is available, users will continue to generate feedback on an ongoing basis and will expect this feedback to be incorporated into the product. Services such as Google Analytics, app download and use statistics, or within-app tracking can provide an in-depth understanding of how and how much a product is being used in a way that is not available for traditional pharmaceutical products. Opportunities also exist to leverage real world data such as health administrative databases to monitor products’ safety on an ongoing basis.^20^

While products are in the post-market surveillance phase, the health landscape, technology, and market will all continue to change, requiring continual evaluation of whether the product is still (1) relevant, useful, and being used; (2) safe to use; and (3) effective both clinically and technologically. Changes in technology will inevitably require frequent iteration of the application so that it can continue to function. This is one of many elements that will comprise the application’s maintenance costs. It is important to continue to evaluate the cost/benefit ratio of the product to determine the merits of the product remaining on the market.

## Conclusion

The standard approach to the evaluation of pharmaceutical products has important limitations when applied to the flexible approach characteristic to the development of many digital products. As such, this approach may potentially deter the efforts to properly evaluate mHealth products as developers, scientists, and health care providers believe that the timelines and approach are not practical. An iterative approach to research that permits more flexibility, rapid evaluation, and changing protocols as technology changes may encourage more evaluations of digital products. Our proposed framework builds on previous strategies to promote improved regulation and evaluation of these products by offering a common set of gating questions and best practices for continuous iteration and ongoing improvement of mHealth solutions.

## Data Availability

No datasets were generated or analysed during the current study.
